# Increased proteasome activator 28 gamma (PA28γ) levels are unspecific but correlate with disease activity in rheumatoid arthritis

**DOI:** 10.1186/1471-2474-15-414

**Published:** 2014-12-08

**Authors:** Melanie Gruner, Anja Moncsek, Stefan Rödiger, Dagmar Kühnhardt, Eugen Feist, Ralf Stohwasser

**Affiliations:** Faculty of Natural Sciences, Brandenburg Technical University Cottbus - Senftenberg, Großenhainer Str. 57, D-01968 Senftenberg, Germany; Department of Rheumatology and Clinical Immunology and Autoinflammatory Reference Centre at Charité, Charité-Universitätsmedizin Berlin, Charitéplatz 1, D-10117 Berlin, Germany; Department of Biochemistry, Charité-Universitätsmedizin Berlin, Charitéplatz 1, D-10117 Berlin, Germany; Department of Hematology and Oncology, Charité-Universitätsmedizin Berlin, Charitéplatz 1, D-10117 Berlin, Germany

**Keywords:** Proteasome activator PA28γ, 20S proteasome, Sandwich ELISA, Microbeads, Autoimmune disorders, Rheumatoid arthritis, Abatacept, Cancer

## Abstract

**Background:**

PA28γ (also known as Ki, REG gamma, PMSE3), a member of the ubiquitin-and ATP-independent proteasome activator family 11S, has been proved to show proteasome-dependent and -independent effects on several proteins including tumor suppressor p53, cyclin-dependent kinase inhibitor p21 and steroid receptor co-activator 3 (SCR-3). Interestingly, PA28γ is overexpressed in pathological tissue of various cancers affecting e. g. breast, bowl and thyroids. Furthermore, anti-PA28γ autoantibodies have been linked to several autoimmune disorders. The aim of this study was to develop and evaluate a novel and sensitive PA28γ sandwich ELISA for the quantification of PA28γ serum levels in patients with cancer and autoimmune diseases for diagnostic and prognostic purposes.

**Methods:**

PA28γ-specific polyclonal antibodies and recombinant His-tagged PA28γ were purified and used to develop a sandwich ELISA for the detection of circulating PA28γ. With this new assay, PA28γ serum levels of patients with various cancers, rheumatoid arthritis (RA), Sjögren’s syndrome (SS), adult-onset Still’s disease (AOSD) and different connective-tissue diseases (CTD) were compared with healthy control subjects. Anti-PA28γ autoantibodies were additionally confirmed using a newly developed microbead assay.

**Results:**

The developed PA28γ sandwich ELISA showed a high specificity with a detection limit of 3 ng/ml. A significant up-regulation of circulating PA28γ was detected in the sera of patients with cancer, RA, SS and CTD. A significant correlation was observed dependent on age as well as anti-PA28γ autoantibody levels with circulating PA28γ protein levels. Furthermore, PA28γ serum levels showed a correlation with disease activity in patients with RA under treatment with the T-cell directed biological compound abatacept according to disease activity score 28 (DAS28) and erythrocyte sedimentation rate (ESR).

**Conclusion:**

The application of PA28γ as a novel biomarker for diagnostic purposes of a specific disease is limited, since elevated levels were observed in different disorders. However, the correlation with disease activity in patients with RA suggests a prognostic value, which needs to be addressed by further studies. Therefore our results show that PA28γ is a useful marker which should be included in studies related to novel treatments, e.g. abatacept.

**Electronic supplementary material:**

The online version of this article (doi:10.1186/1471-2474-15-414) contains supplementary material, which is available to authorized users.

## Background

The proteasome is a multicatalytic protein complex that is essential for the degradation of cytosolic and nuclear proteins. It regulates a number of cellular functions including cell cycle, transcription and antigen processing [1–3]. The barrel-like 20S core particle contains 28 subunits that are arranged in two outer heptameric α-rings and two inner heptameric β-rings which contain the three proteolytic active sites - β1, β2 and β5 exhibit caspase-like, trypsin-like and chymotrypsin-like cleavage preference, respectively [4]. Protein cleavage is highly regulated through controlled substrate entry. Thereby, an activation of latent 20S proteasome by activators is necessary for protein degradation. Whereas polyubiquitinated proteins are degraded through the ATP-dependent 26S proteasome formed by the 20S core and the 19S proteasomal activator (PA700), the 11S regulators (PA28α, PA28β and PA28γ) act in an ubiquitin- and ATP-independent manner [2].

The interferon-γ-inducible PA28α and PA28β subunits form heteroheptamers mainly found in the cytosol and are involved in the production of MHC class I ligands [5]. The so called immunoproteasome including the interferon-γ-inducible β1i, β2i and β5i catalytic subunits is known to generate peptides that are more likely to bind to MHC class I molecules. Thus, immunoproteasomes are more relevant for antigen processing and efficient pathogen combat [3]. Furthermore, proteasomes are reported to be involved in disease prevention due to their complex role in apoptosis and cell cycle. Degradation of pro-apoptotic proteins such as tumor suppressor p53 and cell cycle regulator p21 as well as cleavage of proteins like IκB that leads to the activation of the anti-apoptotic transcription factor NF-κB, reflects the major importance of this enzyme complex [6]. A modified expression and/or activity of constitutive and inducible proteasomes have been reported in several malignant diseases and autoimmune disorders. In Sjögren’s syndrome catalytic subunits are up regulated at the mRNA level while the β1i subunit is deficient in B lymphocytes leading to increased apoptosis resistance after proteasome inhibition [7, 8]. Moreover, circulating proteasomes were measured in serum or plasma samples using ELISA technique demonstrating elevated levels in patients with systemic autoimmune diseases [9] and suggesting a role as an independent prognostic factor in multiple myeloma [10].

Furthermore, the proteasome activator PA28γ (REGγ, Ki, PSME3) that is not interferon-γ-inducible and whose homoheptamers are mainly found in the nucleus was previously regarded to be an activator for the degradation of short peptides [11]. However, former studies reported that PA28γ stimulates also the proteasomal degradation of larger proteins like steroid receptor coactivator-3 (SRC-3/AIB1) [12], cyclin-dependent kinase inhibitor p21 [13, 14], Hepatitis C virus core protein [15], ubiquitin ligase Smad ubiquitination regulatory factor 1 (Smurf1) [16] and insulin transcription activator MAFA [17]. In addition proteasome-independent functions of PA28γ were shown. The activator plays a key role in chromosomal stability during mitosis [18], in the organization of nuclear speckles [19] and during the interaction with nuclear localization protein p30 of Human T-lymphotropic virus type I (HTLV-1) to increase viral spread [20, 21]. Beside these important roles in multiple biological pathways including cell growth and cell cycle regulation, PA28γ is also a mediator of apoptosis. Mdm2-dependent p53 degradation is enhanced by PA28γ acting as a co-activator [22] and p53 activity is regulated through its cellular localization mediated by PA28γ [23]. Due to these functions it seems to be very likely that PA28γ is involved in carcinogenesis. Many studies revealed that PA28γ is overexpressed in different tumor tissues and serum samples of patients with diverse cancers affecting colon [24, 25], breast [26], larynx [27], lung [28], liver [29] and thyroids [30]. Thus, PA28γ interferes with cell cycle, proliferation and invasion in poorly differentiated thyroid carcinoma cells [31]. Interestingly, PA28γ was first identified as the Ki-antigen target of autoantibodies in patients with systemic lupus erythematosus (SLE) [32]. Several subsequent investigators confirmed the detection of PA28γ as an autoantigen in patients with SLE and correlated this antibody response to clinical and serological features including disease activity [33, 34]. These studies demonstrate that there is an urgent need for a rapid and simple robust routine diagnostic test to quantify the serum levels of PA28γ. However, most studies so far have been performed from tissue which is laborious and not quantitative und therefore not applicable for progression monitoring.

The aim of the present study was to develop and to evaluate a quantitative PA28γ sandwich ELISA to clarify its significance for diagnosis and prognosis of different diseases. We suggest that this assay with PA28γ as putative predictive marker is an excellent tool to investigate the complex of diseases (autoimmune and cancer) which are potentially related to alterations in levels of PA28γ. Our first results indicate that PA28γ levels can be reliably measured in serum samples from patients with various diseases like cancer, rheumatoid arthritis (RA) and other autoimmune as well as autoinflammatory disorders. Moreover, PA28γ serum levels seem to correlate with disease activity in RA patients treated with T-cell inhibitor abatacept.

## Methods

### Chemicals and reagents

All chemicals and reagents used were obtained from Carl Roth (Karlsruhe, Germany), Thermo Fisher Scientific (Rockford, USA), Roche Diagnostics (Mannheim, Germany) or Biochrom (Berlin, Germany) unless otherwise stated.

### Patients

All sera were obtained at the Charité University Hospital Berlin, Germany after informed consent. Following patient groups were investigated: 28 patients with various tumors (cancer), 104 patients with rheumatoid arthritis (RA), 34 patients with Sjögren’s syndrome (SS), 15 patients with adult-onset Still’s disease (AOSD), 66 patients with different connective-tissue diseases including polymyositis (PM, n = 18), systemic lupus erythematosus (SLE, n = 37) and undifferentiated connective-tissue disease (UCTD, n = 11) and 20 healthy subjects. Cancer group includes patients with advanced metastatic tumors: 20 subjects with breast cancer, 4 subjects with colon carcinoma, 1 subject with parotis gland cancer, 1 subject with sarcoma, 1 subject with a neuroendocrine tumor and 1 subject with carcinoma of unknown primary. With the exception of 2 cases all cancer patients were under treatment with chemotherapy with or without immuno or hormone therapy.

To study follow-up samples in correlation to disease activity according to DAS28-ESR (disease activity score 28 - erythrocyte sedimentation rate) and CRP (C-reactive protein), 13 RA patients under abatacept were included and sera were obtained before initiation of treatment as well as at week 16 and 24. All RA patients failed respond to methotrexat (MTX) treatment and received abatacept as first-line biologic in combination with MTX.

The study was approved by the local ethic committee at the Charité-Universitätsmedizin Berlin.

### Recombinant antigen production

To obtain protein for standard curve development for sandwich ELISA human PA28γ cDNA was amplified by polymerase chain reaction (PCR) and cloned into pDest17 expression vector following Gateway® Recombinant Cloning Technology (Life Technologies, Darmstadt, Germany). The construct was transformed into *E. coli* BL21(DE3)pLysS and after induction of expression with 2 mM IPTG for 2 h at 30°C bacteria were harvested. Solubilization of precipitated His-tagged PA28γ was performed following a modified method of Ahmed et al. [35]. Frozen pellets were thawed and lysed with lysis buffer (50 mM Tris/HCl pH 8.0, 50 mM NaCl, 1 mM EDTA, complete protease inhibitor). After addition of 300 μg/ml lysozym and 1 mg/ml sodium deoxycholate the suspension was incubated for 30 min on ice and further 15 min at RT after addition of approximately 10 U/ml DNase I and 10 mM MgCl_2_. Insoluble components were pelleted during centrifugation at 17,000 × g for 15 min at 4°C and washed with lysis buffer containing 0.5% Triton-X100 for 10 min at RT. After further centrifugation the pellet was dissolved in lysis buffer containing 8 M Urea for a minimum of 2 h at RT. The soluble protein fraction was dialyzed against 20 mM sodium phosphate buffer pH 7.4 and insoluble impurities were removed by centrifugation (17,000 × g, 15 min). Protein amount was calculated using Pierce BCA Protein Assay Kit and purity was controlled by SDS-PAGE (sodium dodecyl sulfat-polyacrylamid gel electrophoresis).

### Antibodies

The mouse monoclonal antibody raised against amino acids 45–147 of PA28γ of mouse origin was purchased from Santa Cruz Biotechnology (Santa Cruz, USA). Secondary Horse radish peroxidase conjugated goat-anti-rabbit IgG and Cy5-conjugated goat-anti-human IgG were purchased from Dianova (Hamburg, Germany).

For production of polyclonal antiserum directed against PA28γ rabbits were immunized by multiple intradermal injections of a PA28γ specific KLH-coupled peptide representing amino acids 14–28 with citrullinated arginine in amino acid position 6 and 8 (Biogenes, Berlin, Germany). The collected serum (K3946) was precipitated with 40% ammonium sulfate, centrifuged 30 min at 15,000 × g and resuspended pellet was dialyzed against 20 mM sodium phosphate buffer pH 8.0. Clarified (centrifugation at 15,000 × g, 30 min) extract was purified by Protein An affinity column previously equilibrated in the same buffer on Äkta FPLC system (GE Healthcare, Munich, Germany). IgG complexes were eluted with 100 mM Glycin/HCl pH 3.0 and pH was shifted to 8.0 with NaOH. To avoid precipitation an end concentration of 100 mM NaCl was adjusted and pooled IgG fraction was dialyzed against PBS buffer pH 7.4. To get peptide specific antibodies the extract was further purified with an Ultra Link (Thermo Fisher Scientific, Rockford, USA) column containing immobilized PA28γ specific amino acid sequence 14–28 in non-citrullinated form. The chromatography was performed using PBS pH 7.4 as running buffer and 100 mM Glycin/HCl pH 3.0 as elution buffer. The pH of the eluted peptide specific polyclonal rabbit IgG (rb-pIgG) was shifted to pH 8.0 with NaOH and an end concentration of 100 mM NaCl before dialysis against PBS pH 7.4. After clarifying by centrifugation (17,000 × g, 15 min) and protein concentration estimation by photospectrometrical measurement the pooled fractions were adjusted to 0.02% NaN_3_ and stored in Aliquots at −20°C with an end concentration of 500 μg/ml.

### Immunoblotting

For immunoblotting, 30 μg total cell extract of a human carcinoma cell line HT29 (ATCC® HTB-38^TM^) or 1 μg purified recombinant His-PA28γ were separated by SDS-PAGE and transferred onto PVDF membranes. Remaining binding sites were blocked with 5% nonfat dry milk in PBS buffer pH 7.4 containing 0.1% Tween-20 (PBST) for 1 h. Membranes were incubated with crude rabbit hyperimmunserum raised against PA28γ-peptide (1:5,000), eluted fraction of protein A purification step (1:2,500) or rb-pIgG (1:2,500) in PBST for 1 h. Then secondary goat-anti-rabbit IgG antibody coupled with horseradish peroxidase (1:10,000) was added for 1 h. Antibody binding was visualized using ECL solution and released chemiluminescence was detected by using Lumi-Imager F1 (Roche Diagnostics, Mannheim, Germany).

### Sandwich ELISA for quantification of PA28γ in human sera

To quantify the PA28γ amount in human sera an indirect sandwich ELISA was developed. Nunc Polysorp microtiter plates were coated with 100 μl of mouse monoclonal capture antibody diluted 1:500 in 100 mM sodium carbonate buffer pH 9.6 overnight at 4°C. Unbound material was removed with PBST buffer (PBS containing 0.1% Tween 20) and free binding sites were blocked with 300 μl 1 × Roti Block solution (Carl Roth, Karlsruhe, Germany) for 1 h at RT. After one further washing step 100 μl of serum samples diluted 1:2 in PBST were added for 1 h following three washing steps. Wells were incubated with 100 μl detection antibody rb-pIgG (1:250 in PBST) or PBST as negative control for 1 h before washing three times. After addition of 100 μl of horseradish peroxidase (HRP)-conjugated secondary antibody diluted 1:5,000 and incubation for 1 h, the microtiter plate was washed extensively with PBST buffer (three times). HRP activity was determined by adding 100 μl tetramethylbenzidine substrate solution (Sigma-Aldrich, St. Louis, USA) following a 30 min incubation in the dark. Reaction was stopped with 100 μl sulfuric acid stop solution (Sigma-Aldrich, St. Louis, USA) and absorbance was measured at 450 nm with a correction wavelength of 620 nm using the ELISA plate reader Synergy HT (Biotek, Bad Friedrichshall, Germany).

Recombinant His-PA28γ in appropriate dilutions (3.125 to 200 ng/ml) was used for calibration and the PA28γ contents of human sera were calculated by subtracting negative control. The cutoff value was defined as the mean value plus 3-fold standard deviation obtained from 20 healthy controls.

Reproducibility of sandwich ELISA was determined by intra- and inter-assay precision studies and calculated as the mean coefficient of variation (CV). Therefore three sandwich assays with varying His-PA28γ concentrations ranging from 0 to 200 ng/ml were performed on distinct plates at one day and reproduced on 3 different days.

### Microbead assay for determination of PA28γ autoantibody level

Carboxylated poly(methyl methacrylate) microbeads (PolyAn GmbH, Berlin, Germany) were coupled with recombinant His-PA28γ following MES buffer method [36]. The microbeads were immobilized on microtiter plates and incubated with human sera diluted 1:100. After addition of Cy5-conjugated secondary antibody the mean fluorescence intensity (MFI) of the microbeads was analyzed using VideoScan technology [37].

### Statistical analysis

Statistical analyses were performed with IBM SPSS Statistic Version 21.0.0.0 and PKWard [38] using non-parametrical Mann–Whitney U test to compare PA28γ levels in patient groups. The relationship between two variables was calculated using Pearson’s correlation analysis. *P* values less than 0.05 were considered to be statistically significant (*) and less than 0.01 were considered to be highly significant (**).

## Results

### Preparation of recombinant PA28γ and rabbit polyclonal antibodies to PA28γ

**Figure 1:**
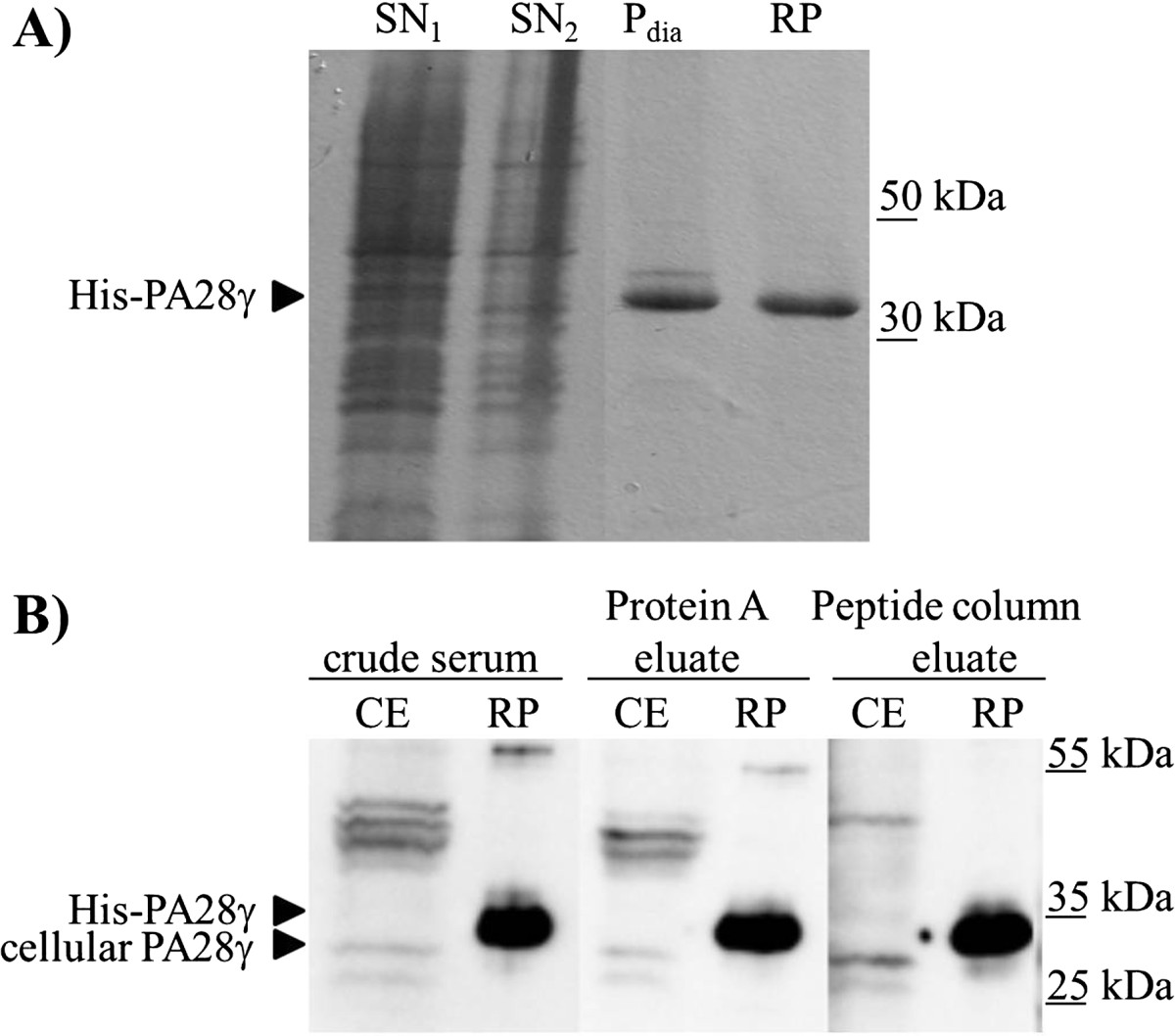
**Preparation of recombinant PA28γ and rabbit polyclonal antibodies to PA28γ. A)** Coomassie stained SDS gel of purified recombinant PA28γ. Supernatant 1 and 2 (SN_1_ and SN_2_) contain the soluble *E. coli* proteins after the first two solubilization steps. Pelleted His-PA28γ precipitate was solved in 8 M urea. After dialysis insoluble His-PA28γ was removed by centrifugation (P_dia_) and soluble recombinant protein (RP) was stored at 4°C. **B)** Immunoblot analysis with anti-PA28γ polyclonal antibody (crude serum, eluate of Protein A purification and eluate of PA28γ specific peptide column). Cellular extract of carcinoma cells (CE, 30 μg) and recombinant His-PA28γ protein (RP, 1 μg) were separated by SDS-PAGE, blotted and incubated with rabbit anti-PA28γ antibody purifications.

To develop a PA28γ sandwich ELISA for evaluating PA28γ content in sera of patients with different diseases a standard protein for quantification was necessary. Therefore we expressed recombinant His-tagged PA28γ in *E. coli* BL21 and precipitated protein was extracted via a purification procedure containing a urea solubilization step. Figure 1A shows the different fractions during purification and the clear soluble His-PA28γ protein that was used to generate the ELISA standard.The immunization of a rabbit with the citrullinated PA28γ-specific peptide revealed one polyclonal hyperimmunserum that formerly was thought to detect citrullinated antigens. Immunoblotting with recombinant un-citrullinated His-PA28γ protein and cell lysates from human colon carcinoma cell line HT29 showed specific interaction of the serum with PA28γ. To reduce background reactivity the serum was affinity purified using a protein A column. PA28γ-specific IgG antibodies were enriched using an Ultra Link column containing the immobilized un-citrullinated peptide immunogen. The specificity of the purified PA28γ antibodies was monitored by western blot analysis (Figure 1B). A 28 kDa band corresponding to cellular PA28γ was detected by the rabbit antiserum in the cellular extract of the carcinoma cells, but there were also other bands corresponding to unspecific absorption or modified PA28γ variants. The background reactivity was reduced by purification of polyclonal serum via protein A and the peptide affinity purification steps. All antibody fractions that were collected during the antibody purification steps showed strong immunoreaction with recombinant His-PA28γ protein. Around 300 μg of highly purified peptide specific polyclonal rabbit PA28γ antibody (rb-pIgG) could be obtained per ml of rabbit hyperimmunserum.

### Sandwich ELISA validation

To validate the reproducibility of the sandwich ELISA the standard protein His-PA28γ was used in varying concentrations ranging from 0 to 200 ng/ml. For intra-assay precision 3 microtiter plates coated with commercial monoclonal PA28γ antibody were incubated with the His-PA28γ solutions and antigen was quantified by using rb-pIgG in combination with a horseradish conjugated secondary anti-rabbit IgG antibody. The mean coefficient of variation (CV) ranged from 0.4% to 31.2% at day 1, from 4.6% to 14.7% at day 2 and from 3.9% to 18.1% at day 3 (Table 1). Accordingly, the inter-assay CVs are represented by 10.9% to 18.1%. The lower limit of PA28γ detection of the sandwich ELISA was found to be 3.125 ng/ml and the assay showed no reactivity with 11S family members PA28α or PA28β (data not shown).

**Table 1:** **Intra- and inter-assay precisions of PA28** γ **sandwich ELISA**

His-PA28γ[ng/ml]								
	0	3.125	6.25	12.5	25	50	100	200
**Intra-assay CV [%], n = 3**								
*day 1*	0.4	10.4	16.7	31.2	19.9	9.3	16.8	10.4
*day 2*	8.7	14.7	7.6	4.6	13.8	7.9	4.9	5.2
*day 3*	18.1	4.9	7.0	16.5	3.9	6.5	9.1	10.9
**Inter-assay CV [%], n = 3**								
*day 1-3*	10.9	10.9	10.9	18.1	15.8	11.9	14.6	13.1

Precisions were calculated as the mean coefficient of variation (CV) of different PA28γ antigen concentrations measured with Sandwich ELISA in 3 independent experiments from duplicate wells at 3 days.

### Circulating PA28γ levels in human serum samples

**Figure 2:**
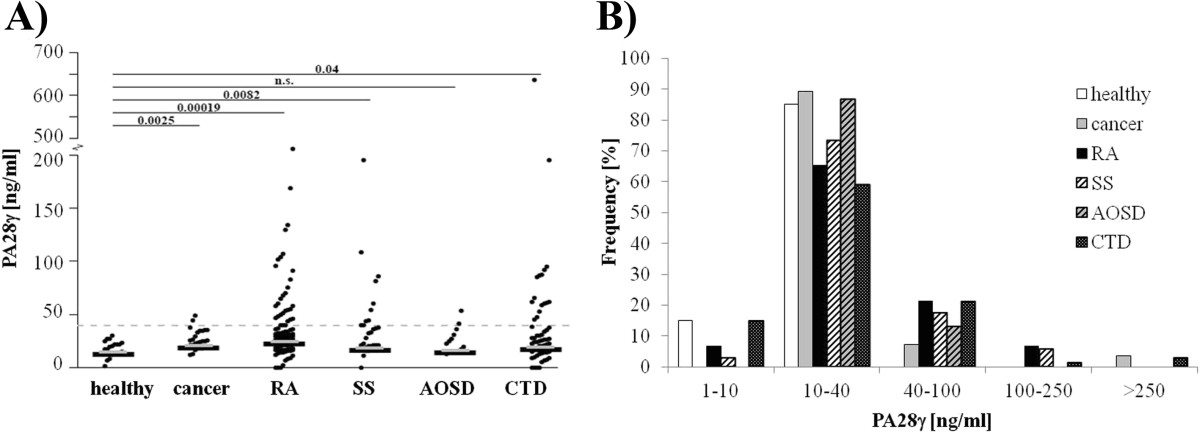
**Circulating PA28γ levels in human sera. A)** Scatter dot plot of circulating PA28γ serum levels measured by sandwich ELISA in patients with cancer (n = 28), rheumatoid arthritis (RA, n = 104), Sjögren’s syndrome (SS, n = 34), adult onset Still’s disease (AOSD, n = 15) and different connective-tissue diseases (CTD, n = 66) compared with healthy controls (n = 20). Dashed line indicates the calculated cutoff value for positive response of 39.8 ng/ml PA28γ as the mean plus 3-fold standard deviation of healthy controls. The results are represented as mean PA28γ levels of duplicate wells. Medians are shown as grey lines. P-values as indicated compared with healthy control. n.s., not significant. **B)** Frequency of different PA28γ serum levels in healthy and pathological subjects.

To determine the levels of circulating PA28γ in sera of patients with different diseases including cancer and autoimmune as well as autoinflammatory disorders a sandwich ELISA using two different PA28γ-specific antibodies was implemented. The cutoff was calculated to be 39.8 ng PA28γ per ml of serum based on the measurement of twenty healthy donors. Summarized, 247 PA28γ serum levels from patients with various autoimmune or malignant diseases were measured and revealed significantly elevated results (median = 25.4 ng/ml, *P* = 0.001) compared with healthy controls (median = 18.1 ng/ml, data not shown). In detail, in patients with Sjögren’s syndrome (SS), a connective tissue disease affecting exocrine glands, 29.4% (10/34) were found to have significantly increased PA28γ levels. Moreover, patients with other autoimmune diseases showed also increased PA28γ levels, in rheumatoid arthritis (RA) 26.9% (28/104), in different connective-tissue diseases (CTD) 25.8% (17/66) and in AOSD (adult-onset Still’s disease) 13.3% (2/15). Of note, increased circulating PA28γ were only detected in 10.7% (3/28) of patients with different types of cancer (Figure 2A).The frequency of different human PA28γ serum levels is displayed in Figure 2B. Most sera showed a PA28γ levels between 10 and 40 ng/ml and were therefore below the calculated cutoff. In up to 21% of patients with autoimmune diseases and cancer, increased PA28γ levels between 40 and 100 ng/ml were measured. Of note, the highest PA28γ concentrations over 100 ng/ml were observed in patients with SS, RA, CTD and cancer, with a maximum level of 637 ng/ml.

The PA28γ levels of cancer patients (median = 246 ng/ml; *P* = 0.003), RA patients (median = 27.8 ng/ml; *P* = 0.0002) and SS patients (median = 21.7 ng/ml; *P* = 0.008) were significantly higher compared with the healthy control group (median = 18.1 mg/ml). All cancer patients termed as PA28γ positive were diagnosed to advanced metastatic breast cancer. In contrast, patients with AOSD, an autoinflammatory disorder, showed no difference with respect to PA28γ levels compared to healthy controls (median = 19.7 ng/ml; *P* = 0.142). Patients with connective-tissue diseases (CTD) showed significantly increased PA28γ levels compared with healthy controls (median = 23.0 ng/ml; *P* = 0.040). In detail, the SLE patient cohort expressed elevated PA28γ protein levels in 9 of 37 cases but the levels were in total not significantly increased compared with healthy controls (median = 18.2 ng/ml; *P* = 0.432). With respect to PA28γ serum levels patients with polymyositis (median = 24.5 ng/ml; *P* = 0.033) and UCTD (median = 30.9 ng/ml; *P* = 0.001) differ in a significant way from healthy controls, respectively (Table 2).

**Table 2:** **Mean PA28** γ **level (ng/ml), standard deviations (SD), minimum (min.) and maximum (max.) values from patients with different diseases**

Disease	n	mean	SD	min.	max.	PA28γpositive	***P*** value
**Healthy**	20	17.5	7.4	1.4	29.6	0.0% (0/20)	
**Cancer**	28	39.6	72.7	11.9	407.8	10.7% (3/28)	**0.003
**RA**	104	37.6	33.5	0.0	205.8	26.9% (28/104)	**0.0002
**SS**	34	37.1	36.1	0.0	195.2	29.4% (10/34)	**0.008
**AOSD**	15	23.8	11.9	12.4	52.9	13.3% (2/15)	0.142
**CTD:**							
*Polymyositis*	18	31.1	21.2	6.7	85.4	22.2% (4/18)	*0.033
*SLE*	37	60.0	127.7	0.0	636.6	24.3% (9/37)	0.432
*UCTD*	11	35.9	15.4	18.4	61.7	36.4% (4/11)	**0.001
**total CTD**	66	48.1	96.8	0.0	636.6	25.8% (17/66)	*0.040

Data over the calculated cutoff are defined as PA28γ positive. *P* values represent significance (*, *P* & 0.05 and **, *P* & 0.01) compared with healthy control group. AOSD; adult-onset Still’s disease, CTD; different connective-tissue diseases, RA; rheumatoid arthritis SLE; systemic lupus erythematosus, SS; Sjögren’s syndrome, UCTD; undifferentiated connective-tissue disease.

### Correlation of circulating PA28γ levels with patients characteristics and disease activity in RA

Of note, PA28γ serum levels seems to be significant negative correlated with age in RA patients (r = −0.223; *P* = 0.032). Furthermore, female RA subjects expressed significantly higher levels of PA28γ (*P* & 0.001) compared with healthy controls, whereas male RA patients differed only marginally (*P* = 0.047). Overall, the difference between male and female RA patients was not significant (*P* = 0.170, data not shown).

The parallel measurement of PA28γ protein levels by sandwich ELISA and anti-PA28γ autoantibody titers by microbead assay in twelve patients with RA revealed a positive correlation (r = 0.648; *P* = 0.023) (data not shown).

To clarify whether circulating PA28γ is bound to intact 20S proteasome complexes a highly specific polyclonal anti-20S rabbit serum was used instead of the polyclonal anti-PA28γ detection antibody in the sandwich assay. Since no measurable signals were obtained, co-capturing could be excluded (data not shown).

**Figure 3:**
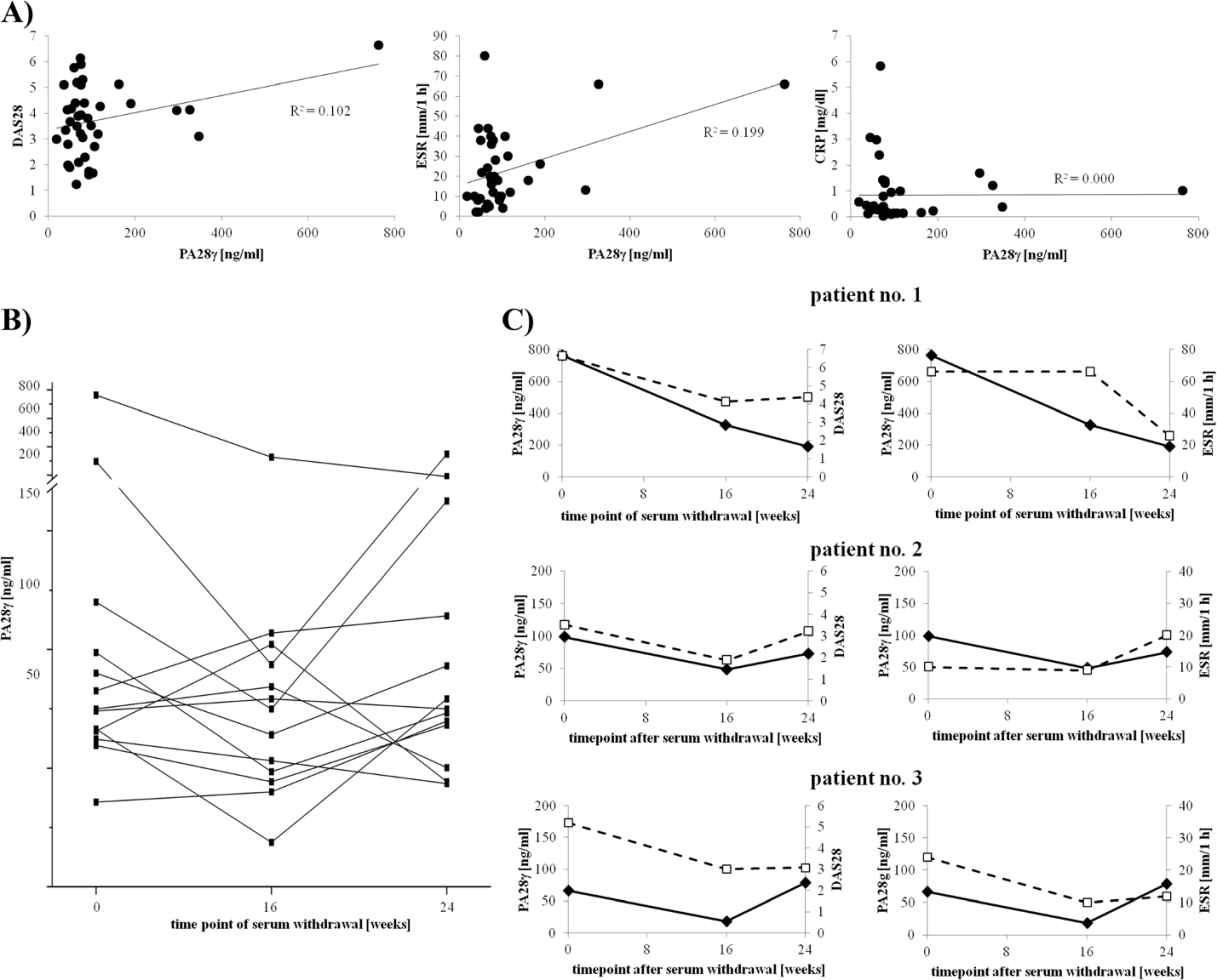
**PA28γ serum levels measured by sandwich ELISA in patients with rheumatoid arthritis treated with Abatacept. A)** Correlation analysis of PA28γ level in sera of 13 RA patients at three time points of serum withdrawal. PA28γ level were compared with disease activity score 28 (DAS28) (r = 0.319, P = *0.047, left), erythrocyte sedimentation rate (ESR) (r = 0.446, P = **0.005, middle) and C-reactive protein (CRP) (r = 0.006, P = 0.971, right). **B)** Course of PA28γ level in Abatacept treated patients with RA. Serum withdrawal was done at indicated time points and the results are represented as mean PA28γ levels of duplicate wells. **C)** Graphical presentation of PA28γ serum level, DAS28 (upper diagrams) and ESR (lower diagrams) of three abatacept treated patients. Black diamonds correspond to PA28γ levels and white rectangles to DAS28 and ESR.

The kinetic of PA28γ serum levels was analyzed in follow-up samples of thirteen RA patients before and under treatment with abatacept after 16 and 24 weeks, respectively (Figure 3). In 61.5% (8/13) of cases the PA28γ levels decreased in the first decade. However, the levels recovered in six of these eight patients after 24 weeks similar to the levels prior to treatment (Figure 3B). Abatacept is a fusion protein composed of the Fc region of IgG1 and the CTLA-4 extracellular domain. This biological compound inhibits the activation of T-cells and thus, has immunosuppressive effects. While proteasome activator family members PA28α and PA28β are interferon-γ-inducible and involved in antigen presentation, the exact role of PA28γ is still unclear. Nevertheless, PA28γ serum levels were compared with disease activity according to DAS28-ESR during abatacept treatment. Of note, a significant correlation with disease activity (r = 0.319, *P* = 0.047) and ESR (r = 0.446, *P* = 0.005), but not with CRP levels under abatacept treatment was confirmed (Figure 3A). In fact, PA28γ levels increased with disease activity and ESR values. Instructive cases over a follow-up period of 6 month are shown in Figure 3C.

## Discussion

In the present study, an indirect sandwich ELISA for the quantification of the proteasome activator 28γ was established. A mono-specific PA28γ antibody recognizing C- and N-terminal regions of the peptide immunogen was purified from polyclonal rabbit serum via a PA28γ peptide column. Recombinant His-PA28γ was expressed in *E. coli*, purified and used as calibration protein. Optimal antibody compositions and dilutions were improved to develop an ELISA method with a sensitivity of 3 ng/ml. To our best knowledge, we describe for the first time elevated PA28γ levels both in sera from patients with different autoimmune and malignant disorders. We showed that the levels differed significantly from the healthy controls. The specificity of the test was confirmed by using recombinant PA28α and PA28β proteins as antigens, which have up to 40% homology with PA28γ [39], but were not detected by the assay.

Several studies suggested PA28γ as a novel biomarker in cancer diseases. Roessler *et al.*[24] detected PA28γ in the circulation and demonstrated high levels of PA28γ in serum samples from patients with colorectal cancer for the first time. They measured PA28γ levels with an immunoassay analogical to a sandwich ELISA, but through time consuming preincubation of serum with biotinylated and digoxigenylated antibodies. Also Chen *et al*. [25] detected an overexpression of PA28γ in tissue of patients with colorectal cancer. Wang *et al.*[26] indicated an overexpression of PA28γ in breast cancer and demonstrated a relation of expression with cancer status and metastasis. Kondo *et al.*[29] mentioned an increase of nuclear PA28γ with the progression of liver disease from hepatitis C virus related chronic hepatitis to liver cirrhosis. These findings suggest PA28γ as target for prevention and/or treatment of hepatocellular carcinoma. Li *et al.*[27] suggested that PA28γ overexpression in laryngeal cancer can promote proliferation and accelerate growth of cancer cells. He *et al.*[28] validated an overexpression of PA28γ in cancer tissue not only on protein level but also on gene level with significant correlation with cancer related genes suggesting a role of PA28γ in tumor development. These facts leads us to assume that PA28γ itself is not only overexpressed in cancer cells but is also detectable in the serum of patients with malignant diseases. In this context, the developed PA28γ sandwich ELISA should clarify if elevated PA28γ levels represent a useful biomarker to distinguish between different disorders or correlates with disease activity.

As a result, our data provided evidence that in healthy subjects the PA28γ serum level is low and ranges from 1.4 to 29.6 ng/ml. In general, a concentration of 10 to 40 ng/ml PA28γ was typical for the majority of all tested patient groups, while increased levels of PA28γ were observed in only certain patients. In fact, nearly all investigated patient groups exhibit significant elevated levels of PA28γ, specifically cancer, SS, PM and UCTD subjects. However, the highest PA28γ levels were observed among patients with rheumatoid arthritis. Of note, AOSD and SLE patients showed no significant elevated PA28γ serum levels compared to healthy controls. Taken together the results indicate that serum levels of PA28γ alone are not useful for differentiation between malignant or systemic autoimmune disorders tested in this study. A qualification of PA28γ serum levels as biomarker to distinguish between different cancers could not be excluded due to low random numbers. However, we were able to demonstrate a difference between healthy and ill subjects. In disease case the PA28γ serum levels showed an average of 39.7 +/− 61.2 ng/ml which is markedly elevated compared with healthy controls (17.5 +/− 7.4 ng/ml). We claim that levels above 39.8 ng/ml can be used as qualitative marker to group patients.

Further, the high frequency of PA28γ elevation in patients with RA was remarkable and was further analyzed in follow-up experiments with active RA before and after initiation of the T-cell directed therapy with abatacept. While PA28α and PA28β are known to be induced by interferon-γ and play a central role in antigen presentation, PA28γ appears not to be majorly involved in immune defense. Surprisingly, a correlation of PA28γ serum levels with DAS28 and ESR were shown. Thus, further studies in larger cohorts of RA patients are of interest to clarify the importance of PA28γ as a new disease activity biomarker. Another interesting observation is the correlation of PA28γ levels with age in RA patients with lower levels in elderly subjects. Recent studies demonstrated that the PA28γ-proteasome system is involved in regulation of aging thru degradation of casein kinase 1 (CK1), which negatively regulates Mdm2. PA28γ depletion in mice lead to p53 accumulation and induced premature aging [40]. The underlying biology of decreasing PA28γ levels in aging RA patients remains unknown and requires further investigations.

In addition to the correlation analyses with well-known biomarkers, PA28γ serum levels were compared with anti-PA28γ autoantibody levels. These autoantibodies were detected in several autoimmune disorders including SLE and SS [41]. Our results indicate a significant connection between PA28γ protein levels and anti-PA28γ autoantibody levels even in RA sera. Circulating PA28γ might exert a pro-inflammatory reaction leading B-cells to the production of misdirected autoantibodies.

It remains unknown, how PA28γ is released into the circulation and whether this is related to increased apoptotic and necrotic cell turnover in patients with cancer and autoimmune disorders. Interestingly, circulating proteins, in particular circulating 20S proteasomes, were also described in various malignant and autoimmune disorders [9, 42, 43]. In this context, a release of cellular 20S proteasome into the serum could lead to the simultaneously passing of bound activator PA28γ. However, our first results revealed no interaction of serological PA28γ and 20S proteasome arguing for an independent release of 20S proteasome and PA28γ but needs further investigations.

## Conclusion

In summary, our findings confirmed that high amounts of PA28γ serum levels are detectable in certain diseases including cancer and autoimmune disorders. The useful potential of PA28γ as novel diagnostic biomarker is limited, due to low specificity. However, the shown correlation with disease activity in RA patients is of further interest. It should be addressed in future studies, whether PA28γ is of prognostic value with relation to pathogenesis in RA.

## Authors’ original submitted files for images

Below are the links to the authors’ original submitted files for images. Authors’ original file for figure 1 Authors’ original file for figure 2 Authors’ original file for figure 3

## Abbreviations

AOSD: Adult-onset Still’s disease
ATP: Adenosine triphosphate
BCA: Bicinchoninic acid
CK1: Casein kinase 1
CRP: C-reactive protein
CTD: Connective-tissue disease
CV: Coefficient of variation
DAS28: Disease activity score 28
ELISA: Enzyme linked immunosorbent assay
ESR: Erythrocyte sedimentation rate
HRP: Horseradish peroxidase
Mdm2: Mouse double minute 2 homolog
MHC: Major histocompatibility complex
mRNA: Messenger ribonucleic acid
PA28γ: Proteasome activator 28 gamma
MTX: Methotrexat
NF-κB: Nuclear factor ‘kappa-light-chain-enhancer’ of activated B-cells
PBS: Phosphate buffered saline
PBST: Phosphate buffered saline plus Tween-20
PCR: Polymerase chain reaction
PM: Polymyositis
RA: Rheumatoid arthritis
rb-pIgG: Peptide specific polyclonal rabbit IgG
RT: Room temperature
SCR-3: Steroid receptor co-activator 3
SDS-PAGE: Sodium dodecyl sulfat-polyacrylamid gel electrophoresis
SLE: Systemic lupus erythematosus
SS: Sjögren’s syndrome
UCTD: Undifferentiated connective-tissue disease.
