# Supplementary figures and images for: Increased proteasome activator 28 gamma (PA28γ) levels are unspecific but correlate with disease activity in rheumatoid arthritis
Source: BMC Musculoskelet Disord. 2014 Dec 8;15:414. doi: 10.1186/1471-2474-15-414 (PMC4295294; doi:10.1186/1471-2474-15-414)

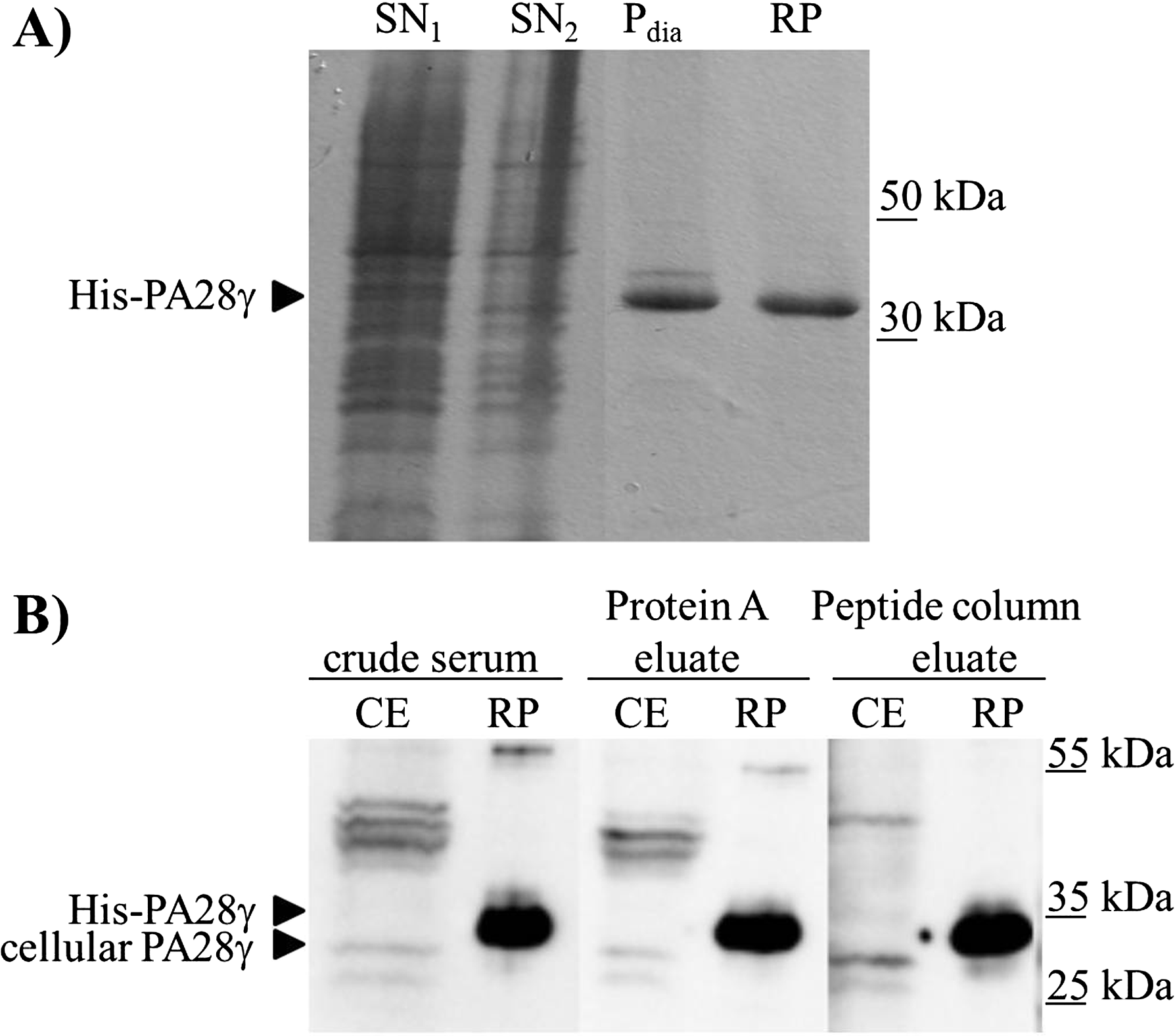

Supplement: Supplementary file 1 — Authors’ original file for figure 1 [file 12891_2014_2360_MOESM1_ESM.tif]

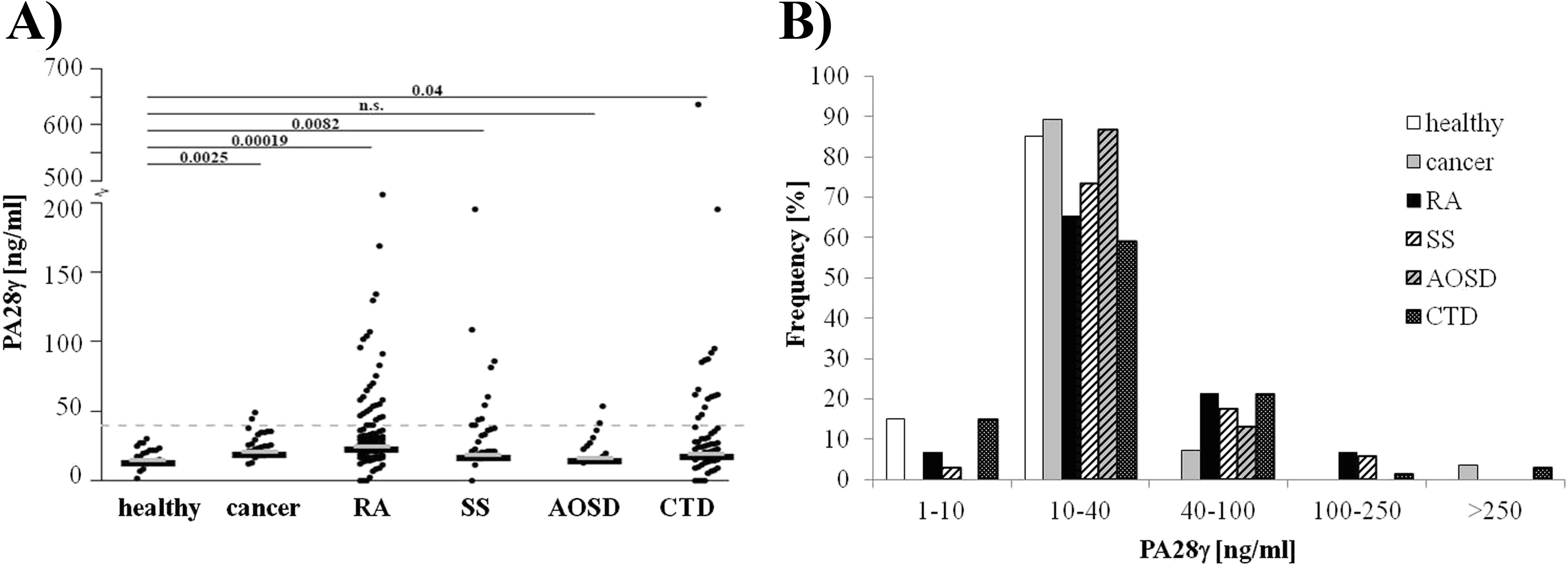

Supplement: Supplementary file 2 — Authors’ original file for figure 2 [file 12891_2014_2360_MOESM2_ESM.tif]

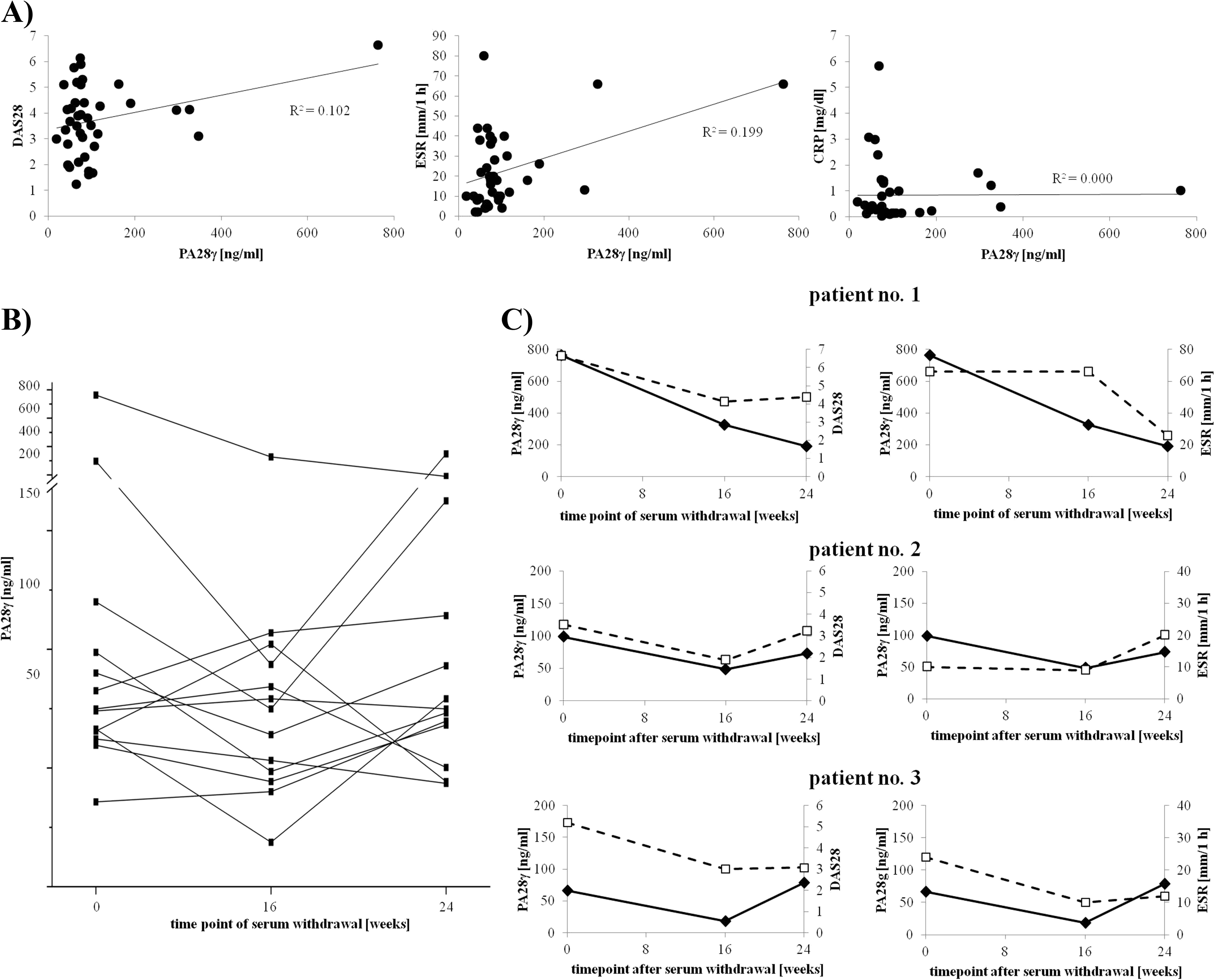

Supplement: Supplementary file 3 — Authors’ original file for figure 3 [file 12891_2014_2360_MOESM3_ESM.tif]
